# In Vitro Synergistic Effects of Antibiotic Combinations Against Multidrug-Resistant *Streptococcus suis* from Diseased Pigs

**DOI:** 10.3390/antibiotics15020136

**Published:** 2026-01-29

**Authors:** Wiyada Chumpol, Kamonwan Lunha, Surasak Jiemsup, Suganya Yongkiettrakul

**Affiliations:** National Center for Genetic Engineering and Biotechnology (BIOTEC), National Science and Technology Development Agency (NSTDA), Pathum Thani 12120, Thailand; wiyada.chu@ncr.nstda.or.th (W.C.); kamonwan.lun@ncr.nstda.or.th (K.L.); surasak@biotec.or.th (S.J.)

**Keywords:** multidrug-resistant *S. suis*, drug combination, synergistic effect, checkerboard assay, time-kill assay

## Abstract

**Background/Objectives:** Multidrug-resistant (MDR) strains of *Streptococcus suis* are increasingly prevalent and present significant challenges in clinical management. Given that the development of new antibiotics is a resource-intensive process and time-consuming, there is an urgent need for alternative therapeutic strategies to address resistance in the short term. One promising approach is the use of combination therapy, which involves pairing potent antibiotics with agents that may be less effective on their own, to enhance therapeutic efficacy and potentially overcome resistance mechanisms. This study aimed to investigate the in vitro antibacterial activity of combining two classes of antibiotics with distinct mechanisms of action—cell wall synthesis inhibitors and protein synthesis inhibitors—against MDR *S. suis* strains isolated from diseased pigs. **Methods:** A total of 36 MDR *S. suis* strains were tested using a microbroth dilution checkerboard assay to determine the minimum inhibitory concentration (MIC) of four cell wall synthesis inhibitors —amoxicillin/clavulanic acid (AMC), ampicillin (AMP), penicillin G (PEN), and vancomycin (VAN)— in combination with four protein synthesis inhibitors —gentamicin (GEN), neomycin (NEO), tilmicosin (TMS), and tylosin (TYL). Time–kill curve assays were conducted to evaluate the in vitro bactericidal activity of synergistic antibiotic combinations (PEN–GEN and AMP–NEO) against Beta-lactam-resistant and Beta-lactam-susceptible MDR *S. suis* strains. **Results:** Checkerboard analysis revealed that penicillin-gentamicin combination exhibited the most effective synergistic activity against the MDR *S. suis* strains (10/19, 52.6%), with ∑FIC values of 0.25–1.06 and MIC reductions from resistant to susceptible levels. Time-kill assays further confirmed the synergistic bactericidal effect of the combination, demonstrating complete bacterial clearance within 6–9 h, markedly rapid bacterial killing compared to monotherapy. **Conclusions**: This study demonstrates that antibiotic combinations, particularly Beta-lactams combined with aminoglycosides, show synergistic activity against pig-isolated *S. suis* MDR strains. The PEN-GEN combination exhibited strong synergistic and bactericidal effects, supporting combination therapy as a potential strategy to address antimicrobial resistance. Further evaluation in diverse strain backgrounds and prudent antibiotic use are essential to confirm efficacy and limit the emergence of antibiotic resistance.

## 1. Introduction

In animal production, antibiotics are widely used for treatment, metaphylaxis, prophylaxis, and growth promotion [1,2]. Such practices exert strong selective pressure on bacterial populations, accelerating the emergence and spread of antimicrobial resistance (AMR) in both veterinary and human medicine [3,4]. *Streptococcus suis* is a major swine pathogen of global concern, responsible for meningitis, septicemia, pneumonia, endocarditis, and arthritis in pigs, and capable of causing severe systemic infections in humans [5]. It also poses a significant public health threat and contributes to substantial economic losses in the pig industry. Due to the economic benefits and therapeutic effectiveness, antibiotics have traditionally been the first line of defense against *S. suis* infections. Despite a 35% reduction in overall sales of medically important antimicrobials in the United States between 2014 and 2023, swine production continues to account for disproportionately high usage with 44% of all those drugs approved for use in food-producing animals [6].

Of particular concern is the continued use of antibiotics critical to human medicine, such as macrolides and fluoroquinolones, which intensifies selection pressure and fosters the emergence of multidrug-resistant (MDR) bacteria, those resistant to at least one agent in three or more different antimicrobial classes, as a result of the widespread and, at times, inappropriate use of antibiotics across both human and veterinary sectors [3,4,7]. In our previous study by Lunha and colleagues [7], over 90% of *S. suis* isolates from diseased pigs in Thailand were resistant to several commonly used antibiotics for the treatment of streptococcal infections in humans and pigs, including tetracyclines, aminoglycosides, fluoroquinolones, and macrolides. Over half of the isolates were also resistant to sulfonamides and cephalosporins. These resistance levels mirror global AMR trends, reflecting the broader challenge of AMR in veterinary medicine [8,9,10]. Moreover, an increasing level of antibiotic resistance to third generation antibiotics has been reported worldwide [10,11,12], which has been recognized as a global problem for public health.

The increasing prevalence of MDR *S. suis* demand timely and practical interventions. While novel effective antibiotics are urgently needed, the lengthy drug development process highlights the importance of immediate alternatives. Combination therapy with existing antibiotics has emerged as a promising strategy to enhance treatment efficacy and address AMR in the short term. This is particularly relevant for *S. suis* infections, where effective treatment options are limited. Several studies have demonstrated the potential of synergistic combinations against *S. suis*, including ampicillin-apramycin, tiamulin-spectinomycin, and ceftriaxone-penicillin [13]. Notably, the novel pleuromutilin derivative GML-12, in combination with tetracycline, showed synergistic or additive effects, suggesting promise for future therapeutic development [14]. These findings support the rational use of combination therapy as an alternative or complementary strategy to monotherapy, particularly in cases involving resistant strains. Despite previous reports describing antibiotic combinations against *S. suis*, available evidence remains limited with respect to recently collected multidrug-resistant (MDR) clinical isolates from different geographic settings. The resistance landscape of *S. suis* has evolved substantially over the past decade, with regional variation in antimicrobial usage shaping distinct resistance phenotypes.

In this context, the present study aimed to identify effective antibiotic drug combinations for MDR *S. suis* isolates collected in Thailand between 2018–2020, a period marked by emerging resistance pattern and increasing resistance to frontline agents such as penicillin, gentamicin, and macrolides. The selection of antibiotic combinations was informed by our previous surveillance study [7], in which pairwise antimicrobial susceptibility data were analyzed using Pearson’s correlation analysis to explore relationships between resistance profiles across antibiotic classes, revealing significant negative correlations between selected drug pairs. Based on these previous findings, specific antibiotic combinations were selected for experimental evaluation using a microbroth dilution checkerboard assay, followed by time-kill assays to further characterize inhibitory and bactericidal effects under combination conditions. By providing functional in vitro data on antibiotic combinations tested against a contemporary, region-specific panel of MDR *S. suis* isolates, this study extends earlier combination studies conducted in different geographic and provides experimental evidence on synergistic drug interactions relevant to the current resistance landscape of this zoonotic pathogen.

## 2. Results

### 2.1. MIC Distribution and Antibiotic Susceptibility Profile of MDR S. Suis

**Figure 1:**
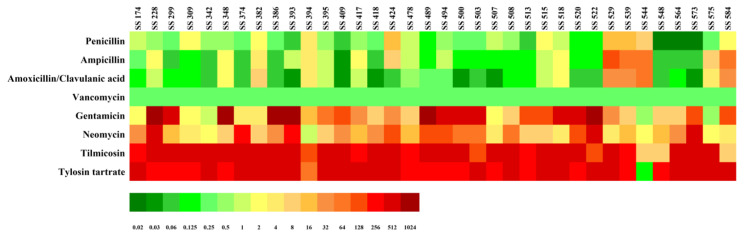
Minimum inhibitory concentration (MIC) results for multidrug-resistant *S. suis* isolates (*n* = 36). MIC values are represented on a color scale, with green indicating low MICs and red indicating high.

The MIC distributions of four cell wall synthesis inhibitors (AMC, AMP, PEN, and VAN) and four protein synthesis inhibitors (GEN, NEO, TMS, and TYL) against 36 MDR *S. suis* strains were presented in Table 1 and Figure 1. High resistance rates were observed for the protein synthesis inhibitors, with 97.3% for TYL, 91.9% for TMS, 59.5% for GEN, and 48.6% for NEO. In contrast, VAN and AMC remained the most effective cell wall synthesis inhibitors, with susceptibility rates of 100.0% and 81.1%, respectively. Among the 36 MDR *S. suis* isolates, AMP and PEN showed moderate susceptibility rates of 54.1% and 45.9%, and an increased proportion of intermediate susceptibility was observed for both drugs (18.9% for PEN and 13.5% for AMP).

### 2.2. Synergistic Drug Interaction

A total of 16 antibiotic combinations were generated by pairing each of the four cell wall synthesis inhibitors (AMC, AMP, PEN, and VAN) with each of the four protein synthesis inhibitors (GEN, NEO, TMS, and TYL), and these combinations were tested for synergistic activity against various MDR *S. suis* (Figure S1).

The checkerboard assay was conducted using combinations of PEN with GEN, NEO, TYL, and TMS against 19, 22, 23, and 14 MDR *S. suis* strains, respectively (Table 2, Table S1). Among these, the PEN-GEN exhibited the highest number of in vitro synergistic interactions, with synergy observed in 10 out of 19 strains (52.6%). The ∑FIC values ranged from 0.25 to 1.06, indicating strong synergy to indifference. No antagonistic interaction was observed for the PEN-GEN. In synergistic cases, the MICs of PEN and GEN were reduced by 8–33-fold and 4–8-fold, respectively, compared to monotherapy. This combination was particularly effective against MDR *S. suis* strains that were non-susceptible to PEN, GEN, or both. Synergistic effects were observed in PEN-non-susceptible strains (3/5, 60.0%), GEN-non-susceptible strains (3/8, 37.5%), and both PEN and GEN non-susceptible strains (4/6, 66.7%). Additionally, partial synergism was detected in PEN-non-susceptible strains (2/5, 40%) and GEN-non-susceptible strains (4/8, 50%).

The PEN-NEO combination was tested against 22 MDR *S. suis* strains and exhibited the highest proportion of partial synergy (9/22, 40.9%), while full synergy and indifference were observed at the rates of 31.8% and 27.3%, respectively. No antagonistic interactions were detected. The ∑FIC values ranged from 0.19 to 1.06, indicating a spectrum from strong synergy to indifference. In synergistic cases, the MICs of PEN and NEO were reduced by 4–13-fold and 4–8-fold, respectively, compared to their individual use. Notably, partial synergy was predominant among isolates non-susceptible to both PEN and NEO (3/4, 75.0%). Among all synergy cases, the most frequent pattern was found in MDR *S. suis* strains that were susceptible to PEN but non-susceptible to NEO (3/7, 42.9%).

The checkerboard assay results for PEN-TYL and PEN-TMS combinations, tested against 23 and 14 MDR *S. suis* strains respectively, revealed predominantly indifferent interactions in 78.3% and 78.6% of the cases.

The synergistic interactions between AMP and GEN, NEO, TYL, and TMS were assessed against 22, 20, 10, and 15 MDR *S. suis* strains, respectively (Table 3, Table S2). Among the combinations tested against 20 MDR *S. suis* strains, AMP-NEO showed the highest proportion of synergistic interactions, with synergy (8/20, 40.0%) and partial synergy (10/20, 50%). Notably, synergy was observed in AMP-non-susceptible strains (4/13, 30.8%), NEO-non-susceptible strains (3/5, 60.0%), and AMP-NEO co-non-susceptible strains (1/2, 50.0%). Among the synergistic cases, the ∑FIC values ranged from 0.31 to 0.50 and the MICs of AMP and NEO were reduced by 4–16-fold and 4-fold, respectively when used in combination. In comparison, AMP combined with GEN, TMS, and TYL showed lower synergy rates of 22.7%, 10.0%, and 20.0%, respectively. The combination of AMP with TYL predominantly resulted in indifferent effects (7/10, 70.0%).

The synergistic effects of AMC in combination with GEN, NEO, TMS, and TYL were evaluated against 21, 19, 10, and 20 MDR *S. suis* strains, respectively (Table 4, Table S3). The results showed that the AMC exhibited partial synergistic activity when combined either with GEN or NEO while no clear synergistic effect was observed for combinations with TYL or TMS. The AMC-TMS combination resulted predominantly in indifferent interactions, with 90% of strains showing no enhanced bactericidal effect. The AMC-TYL combination showed variable outcomes, ranging from partial synergy in 50% of strains to indifference in 60%, suggesting inconsistent interaction across the tested isolates.

The checkerboard assay results for VAN-based combination tested against four MDR *S. suis* strains (Table 5, Table S4) revealed no clear synergistic effects. Partial synergy to indifference was observed when VAN was combined with either GEN or NEO, indicating limited enhancement of antibacterial activity. In contrast, the combinations of VAN with TYL and TMS demonstrated complete indifference across all tested strains.

### 2.3. Effect of Drug Combinations on Bacterial Cell Growth

To validate the synergistic effects observed in the checkerboard assays, time-kill studies were performed using representative *S. suis* strains SS394 (resistant to AMP, GEN, PEN, NEO, TMS, and TYL) and SS500 (resistant to GEN, NEO, TMS, and TYL, but susceptible to Beta-lactam). SS394 was selected to assess combinations against Beta-lactam-resistant MDR strains, while SS500 served as a comparison for Beta-lactam-susceptible MDR strains. Both strains were chosen based on their resistance profiles and observed synergistic interactions in checkerboard assays.

The drug combinations PEN-GEN and AMP-NEO were chosen for the time-kill studies because they demonstrated notable synergistic activity in the checkerboard analysis. Bacterial viability was monitored over 24 h, and synergy was defined as a ≥ 2 log_10_ CFU/mL reduction compared to the most active single agent at 24 h. Two treatment conditions were evaluated: (1) monotherapy using MIC values from single-drug testing, and (2) combination therapy using MIC values based on reduced MICs from checkerboard assays.

**Figure 2:**
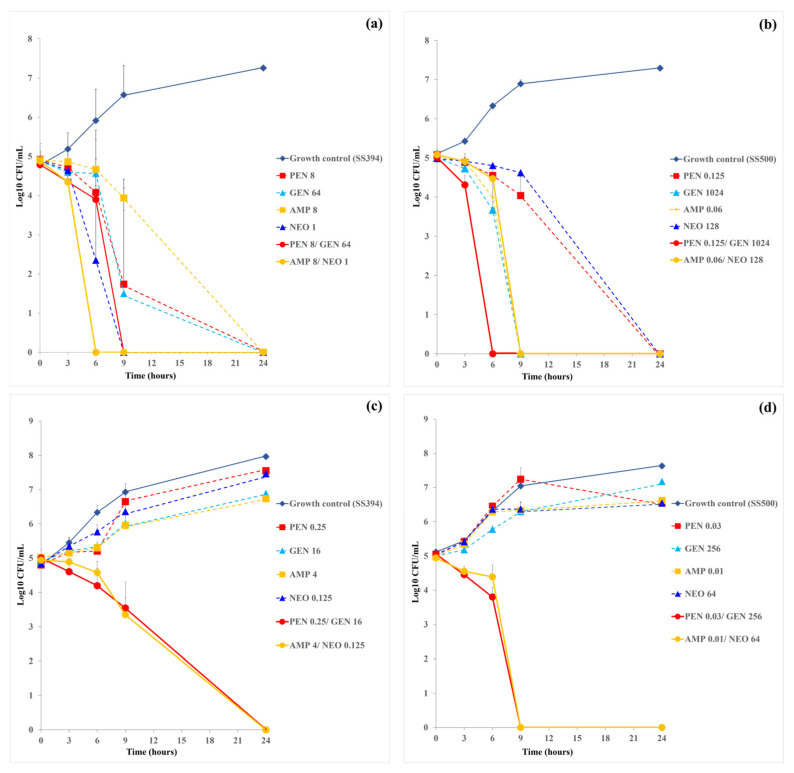
Time-kill curves for single-drug and two-drug combinations at actual MICs and synergistic concentration against multidrug resistance *S. suis* strains SS394 (**a**,**c**) and SS500 (**b**,**d**). Each panel illustrates the time-kill kinetics of penicillin (PEN), gentamicin (GEN), and the PEN-GEN combination (**a**,**b**), or ampicillin (AMP), neomycin (NEO), and the AMP-NEO combination (**c**,**d**). Data are presented as mean ± SD calculated from two independent biological replicates.

In *S. suis* SS394, monotherapy with AMP (8 µg/mL) achieved complete killing by 24 h, while PEN (8 µg/mL), GEN (64 µg/mL), and NEO (1 µg/mL) achieved killing by 9 h. The PEN-GEN combination eradicated bacteria within 9 h, and the AMP-NEO combination achieved complete killing more rapidly, by 6 h (Figure 2a). Under the combination MIC conditions derived from checkerboard assays, both PEN-GEN and AMP-NEO combinations required lower drug concentrations to achieve bacterial killing. In these settings, bacterial counts began to decline within 6 h and reached undetectable levels by 24 h, whereas persistence was observed with monotherapies, confirming their synergistic potential (Figure 2c).

In *S. suis* SS500, time-kill assays under monotherapy conditions revealed that PEN and NEO alone achieved complete killing by 24 h, while AMP and GEN reached bactericidal levels earlier, by 9 h. The PEN-GEN combination resulted in complete bacterial eradication as early as 6 h, indicating a strong synergistic effect (Figure 2b). When tested under the combination MIC condition, PEN-GEN combination maintained rapid bactericidal activity, eliminating viable bacteria by 9 h. In contrast, AMP-NEO combination exhibited a complete bacterial killing within 24 h, suggesting a slower or less potent synergy in this strain (Figure 2d). Compared to SS394, SS500 exhibited a more pronounced and rapid response to PEN-GEN combination therapy.

## 3. Discussion

Emergence and spread of antibiotic resistance in *S. suis* pose a serious threat worldwide, highlighting the urgent need for more effective treatment strategies. Antibiotic combination therapy offers a promising approach, as it can enhance treatment efficacy against resistant *S. suis* strains and help overcome the broader limitations of monotherapy in managing severe bacterial infections [20]. In this context, combination therapies involving already approved drugs are particularly attractive, as they can be developed more rapidly and at lower cost than new chemical entities, making them highly relevant in clinical settings.

The use of antibiotic combinations provides important therapeutic advantages. By pairing agents with complementary mechanisms of action, treatment efficacy can be enhanced while reducing the likelihood of resistance development. Combination therapy can also broaden the antibacterial spectrum, allowing coverage of diverse pathogens [21,22,23,24,25,26,27]. Classic examples of antibiotic synergy illustrate how complementary mechanisms of action enhance antibacterial activity. Beta-lactams combined with Beta-lactamase inhibitors such as clavulanic acid or tazobactam restore antibacterial activity by preventing enzymatic degradation of the Beta-lactam agent [22]. Similarly, synergistic activity observed with PEN-GEN against *Enterococcus faecalis* arise from Beta-lactam-mediated cell-wall disruption, which facilitates intracellular penetration of GEN to its ribosomal target [24]. In *Streptococcus pneumoniae*, LEV-CRO has been shown to enhance bacterial killing through coordinated inhibition of DNA replication and cell-wall synthesis, respectively [26]. Importantly, such synergistic interactions may allow effective bacterial inhibition or killing at drug concentrations below individual clinical susceptibility breakpoints, potentially reducing selective pressure on single agents.

Mechanistically, Beta-lactams such as PEN and AMP exert their antibacterial activity by specifically inhibiting the transpeptidase that catalyzes the cross-linking of peptidoglycan in the final step of cell wall biosynthesis [28]. Whereas aminoglycosides such as GEN and NEO inhibit protein synthesis by binding, with high affinity, to the A-site on the 16S rRNA within the 30S ribosomal subunit [29]. This strong bactericidal action can lead to disruption of the bacterial outer membrane, resulting in leakage of intracellular proteins and impaired bacterial growth. Such complementary mechanisms of action explain the enhanced antibacterial efficacy observed when Beta-lactams and aminoglycosides are used in combination [30,31].

Building on our earlier research, we found that *S. suis* strains were often highly resistant to antibiotics that inhibit protein synthesis, with increased non-susceptibility to Beta-lactams and cephalosporins [7]. In that study, pairwise correlation analysis revealed varying degrees of correlation between resistance to different antibiotics. A significant negative correlation was observed, particularly between protein synthesis inhibitors and cell wall synthesis inhibitors. NEO showed a significant negative correlation with PEN and AMP, while GEN was also significantly negatively correlated with PEN. In veterinary medicine, however, resistance to aminoglycosides is common, and there is a growing trend of intermediate susceptibility and resistance to Beta-lactams. To maximize the utility of existing antibiotics and reduce the risk of further resistance, effective strategies to repurpose antibiotics considered inactive in current veterinary settings also warrant investigation. Hence, this follow-up study assessed the potential synergistic effects of combining a cell wall synthesis inhibitor with a protein synthesis inhibitor against MDR *S. suis* strains isolated from pigs.

While the synergistic effect between cell wall synthesis inhibitors and aminoglycosides has been well established, aminoglycosides, especially GEN, have been recommended for use in combination therapy with agents such as PEN, AMP, and CRO [25,26]. The PEN-GEN combination demonstrated enhanced bactericidal activity against Enterococci and endorsed by the American Heart Association for the treatment of infective endocarditis [27,32]. Our study on MDR *S. suis* strains showed that the PEN-GEN combination yielded the highest synergy rate (∑FIC of <0.5, 52.6%) among all combinations tested. Notably, synergy was observed even in non-susceptible *S. suis* strains, including PEN/AMP/GEN-resistant strain SS394 and GEN-NEO-resistant strain SS500, with 100% synergy and MIC reductions of up to 33-fold for PEN and 8-fold for GEN, indicating a stronger enhancement of PEN activity. Synergy was also evident in PEN-non-susceptible strains, suggesting that GEN may help restore PEN efficacy. However, the effect appeared to be primarily reliant on PEN action.

Time-kill assays confirmed rapid bactericidal effects, achieving complete killing within 6 h, demonstrating a faster bactericidal effect compared to monotherapy. Moreover, a lower concentration of the PEN-GEN combination was sufficient to achieve complete bacterial killing within 24 h. Interestingly, SS500 showed a faster and more pronounced response to PEN-GEN combination therapy than SS394, highlighting strain-specific variability in susceptibility and synergistic interaction. These results emphasize the need to select antibiotic combinations based on individual resistance profiles. Interestingly, PEN-GEN synergistic effects also observed in PEN-susceptible strains, suggesting benefits for both MDR (R→S transition) and susceptible (S→S transition) backgrounds. These findings could support the hypothesis that PEN, as a cell wall synthesis inhibitor, may facilitate increased uptake or intracellular access of GEN, thereby amplifying the bactericidal effect of the combination. Overall, PEN appeared to play a more prominent role than GEN in driving synergy, though the extent of the effect remained strain dependent.

The AMP-GEN combination has previously demonstrated strong synergistic efficacy, showing 80.0% synergy against MDR *Staphylococcus aureus* [33]. In *S. suis*, our study found that AMP showed the broadest and most consistent synergistic activity among the cell-wall synthesis inhibitors evaluated against the MDR *S. suis* strains tested, particularly when paired with NEO. The AMP-NEO combination achieved the highest synergy rate (40.0%) across multiple MDR strains and remained effective in strains that were non-susceptible to AMP and/or NEO. By contrast, AMC combinations displayed moderate synergy, with AMC-NEO and AMC-GEN combinations yielding 21.1% and 23.8% synergy, respectively, while VAN exhibited the lowest overall interaction, limited to a single VAN-NEO-susceptible strain. This comparison highlights AMP-based combinations, especially AMP-NEO, as promising candidates for enhancing treatment efficacy against MDR *S. suis*. Such activity may be related to the capacity of β-lactams to partially overcome aminoglycoside resistance mediated by enzymes including *aac(6′)-aph(2″)* or *ant(6)-Ia* [4]. Future genomic analysis of MDR isolates will be essential to validate these mechanistic hypotheses and to link synergy patterns with specific resistance determinants.

However, the distinct time-kill profiles observed between SS394 and SS500 suggest that strain-specific resistance backgrounds may influence the kinetics of bactericidal activity under combination therapy, even though both strains ultimately exhibited bactericidal responses to the AMP–NEO combination. Given the limited number of strains tested, these findings should be interpreted with caution, and further evaluation using a broader range of strains is recommended.

Compared to AMP-based combinations, AMC exhibited lower or less consistent synergistic potential. For instance, the AMP-NEO combination showed higher synergy (40%) and partial synergy (50%), making it more promising than AMC-NEO. These findings suggest that, in combination with NEO, AMC exhibited some synergistic potential; however, its efficacy was generally inferior to AMP. This difference may be attributed to the distinct pharmacodynamic profiles of AMC and AMP or the limited contribution of clavulanic acid to synergy in this bacterial context. Further mechanistic studies are needed to clarify the role of Beta-lactamase inhibition in these interactions.

Macrolides such as tylosin (TYL) and tilmicosin (TMS) inhibit bacterial protein synthesis by binding to the 50S ribosomal subunit, thereby blocking peptide chain elongation and halting protein production [4]. They are commonly used on pig farms to prevent outbreaks of infections caused by Gram-negative bacteria, primarily from *Lawsonia intracellularis*, *Brachyspira hyodysenteriae*, *Mycoplasma* sp., and *Actinobacillus pleuropneumoniae* [34]. TYL has been investigated for its effects on *S. suis*, particularly regarding biofilm formation and virulence [35,36]. However, the clinical efficacy of TYL against *S. suis* is limited due to high resistance rates. Several studies have highlighted the growing concern of resistance to TYL and TMS in *S. suis* infections across various regions, including Asia, Europe, and North America [4,37,38]. Surveillance data from Thailand between 2018 and 2020 revealed 97.3% and 91.9%, of *S. suis* isolates from diseased pigs were resistant to TYL and TMS, respectively [7]. In the present study, neither TYL nor TMS demonstrated synergistic effects when combined with cell wall synthesis inhibitors against tested MDR *S. suis* strains. The high rates of resistance to macrolides and the limited synergistic benefits observed with macrolide-containing combinations, specifically TYL and TMS, highlight the need for prudent antibiotic stewardship. The high prevalence of TYL and/or TMS resistance in *S. suis* is likely driven by the horizontal transfer of resistance genes among bacterial populations [39,40]. Therefore, the use of these macrolides for treating *S. suis* infections should be approached with caution, and susceptibility testing is strongly recommended prior to their use. Moreover, overuse of macrolides in livestock production likely contributes to this resistance burden, reinforcing the importance of surveillance and rational use of antibiotics to preserve therapeutic efficacy.

In summary, while this study demonstrates synergistic effects of Beta-lactam–aminoglycoside combinations against multidrug-resistant *S. suis*, the findings are limited to in vitro assays (MIC determination, checkerboard, and time-kill experiments). The observed synergistic effects were strain dependent, indicating variability in response among *S. suis* isolates and highlighting the need to examine a broader and more diverse strain panel to determine the broader applicability of these interactions. Despite these limitations, the in vitro results offer meaningful insights into the potential of certain antibiotic combinations and provide a basis for identifying promising candidates for further investigation toward therapeutic application. To strengthen the translational relevance, in vivo pharmacokinetic and pharmacodynamic studies in suitable animal models are recommended to verify efficacy and safety under physiological conditions.

## 4. Materials and Methods

### 4.1. Bacterial Strains

The antimicrobial susceptibility profiles of *S. suis* strains isolated from diseased pigs, provided by Asst. Prof. Dr. Pornchalit Assavacheep, Department of Veterinary Medicine, Faculty of Veterinary Science, Chulalongkorn University (Bangkok, Thailand), were previously analyzed and reported elsewhere [7]. In that study, Pearson’s pairwise correlation analysis was applied to assess relationships between antimicrobial resistance profiles across different antibiotic classes, revealing statistically significant negative correlations between selected antibiotic classes. Based on these previously published correlation analyses and the identified inverse resistance patterns [7], 36 multidrug-resistant (MDR) *S. suis* strains were selected for antibiotic combination testing in the present study. Antibiotic combinations were evaluated using checkerboard microdilution and time-kill assays. *S. pneumoniae* ATCC 49619 was used as a quality control strain. *S. suis* serotypes were determined by multiplex PCR using primers grouped into four sets covering major serotypes: set 1 (serotypes ½, 1, 2, 3, 7, 9, 11, 14, and 16); set 2 (serotypes 4, 5, 8, 12, 18, 19, 24, and 25); set 3 (serotypes 6, 10, 13, 15, 17, 23, and 31); and set 4 (serotypes 21, 27, 28, 29, and 30) [7].

### 4.2. Antibacterial Agents

Based on pairwise resistance correlation data [7], eight antibiotics with distinct modes of action were selected for susceptibility and synergistic testing against selected *S. suis* strains. These included cell wall synthesis inhibitors—amoxicillin/clavulanic acid (AMC), ampicillin (AMP), penicillin G (PEN), and vancomycin (VAN); and protein synthesis inhibitors—gentamicin (GEN), neomycin (NEO), tilmicosin (TMS), and tylosin (TYL). All antibiotics were obtained from Sigma Aldrich Co., Ltd. (Saint Louis, MO, USA).

### 4.3. Antimicrobial Susceptibility Tests

The minimum inhibitory concentrations (MICs) of eight individual antibacterial agents were determined for all 36 MDR *S. suis* strains using the broth microdilution method, following CLSI guidelines [15]. Overnight cultures grown on Columbia blood agar (Clinical Diagnostics Ltd., part, Bangkok, Thailand) were suspended in cation-adjusted Mueller-Hinton broth with 5% lysed horse blood (CAMHB-LHB, Oxoid, Thermo Fisher Scientific Co., Ltd., Lenexa, KS, USA) and adjusted to a 0.5 McFarland standard (~10^8^ CFU/mL). The bacterial suspension was then diluted to a final inoculum of ~10^6^ CFU/mL. Two-fold serial dilutions of each freshly prepared antibiotic were dispensed into 96-well microtiter plates (Thermo Fisher Scientific Inc., Roskilde, Denmark), followed by inoculation with the bacterial suspension. After 24 h of incubation at 37 °C in 5% CO_2_, plates were examined for turbidity using a Sensititre™ Manual Viewbox (Thermo Fisher Scientific Inc., West Sussex, UK). The MIC was defined as the lowest concentration of antibiotics that completely inhibited visible bacterial growth. Susceptibility interpretations (susceptible (S), intermediate (I), and resistant (R)) were based on breakpoints from CLSI [15], FDA [16], and previously reported data [17,18,19] (Table S5). In this study, strains were classified as either susceptible (S) or non-susceptible (NS), with the latter category including both intermediate and resistant strains.

### 4.4. Checkerboard Microdilution Assay

The potential synergistic effects between cell wall synthesis inhibitors and protein synthesis inhibitors were evaluated using a checkerboard microdilution assay, modified from previously described methods [13,41], and performed in 96-well microtiter plates. Antibiotic combinations consisted of individual cell wall synthesis inhibitors-penicillin G (PEN), ampicillin (AMP), amoxicillin/clavulanic acid (AMC), or vancomycin (VAN)-each paired with a single protein synthesis inhibitor-gentamicin (GEN), neomycin (NEO), tilmicosin (TMS), or tylosin (TYL).

Checkerboard assays were performed in 96-well plates. Antibiotic combinations were tested using six predefined concentrations for each drug: 1/8 × MIC, 1/4 × MIC, 1/2 × MIC, 1 × MIC, 2 × MIC, and 4 × MIC. Each well was inoculated with bacterial suspension at ~10^6^ CFU/mL. Positive growth controls consisted of wells without antibiotics, while negative controls included wells containing only CAMHB and antibiotic dilutions without inoculum. Synergistic activity was determined by the absence of visible growth after 24 h incubation at 37 °C in 5% CO_2_. The fractional inhibitory concentration index (∑FIC), which quantifies the interaction between two antibacterial agents when tested in combination, was calculated to assess the interaction between antibiotic pairs as follows:

$$\sum \mathrm{FIC}=\frac{\mathrm{MIC}\;\mathrm{of}\;A\;\mathrm{in}\;\mathrm{combination}}{\mathrm{MIC}\;\mathrm{of}\;A\;\mathrm{alone}}+\frac{\mathrm{MIC}\;\mathrm{of}\;B\;\mathrm{in}\;\mathrm{combination}}{\mathrm{MIC}\;\mathrm{of}\;B\;\mathrm{alone}}$$

Based on the criteria defined by Aranda et al. (2019) [42], the results were interpreted as follows: total synergism (∑FIC ≤ 0.5), partial synergism (0.5 < ∑FIC ≤ 0.75), indifference (0.75 < ∑FIC ≤ 2), or antagonism (∑FIC > 2).

### 4.5. Time-Killing Curves of Synergistic Combinations

Time-kill curve assays were conducted in duplicate to evaluate the in vitro bactericidal activity of selected antibiotic combinations over a 24-h period, following previously described method [43]. The MDR *S. suis* strains were selected based on their resistance profiles and strong synergistic interactions identified in the checkerboard microdilution assay (i.e., the lowest ∑FIC values). Two representative *S. suis* strains were used: SS394, which is resistant to AMP, GEN, PEN, NEO, TMS, and TYL, and SS500, which is resistant to GEN, NEO, TMS, and TYL, but susceptible to Beta-lactam. SS394 was selected to assess antibiotic combinations against Beta-lactam resistant MDR strains, while SS500 served as a comparator representing Beta-lactam-susceptible MDR strains. The drug combinations PEN-GEN and AMP-NEO were chosen for the time-kill studies because they demonstrated notable synergistic activity in the checkerboard analysis.

Briefly, *S. suis* was cultured on Columbia blood agar (BA) and incubated at 37 °C for 24 h in 5% CO_2_. A 0.5 McFarland standard was prepared from a single colony. The bacterial suspension was diluted to 10^6^ CFU/mL in CAMHB. The inoculum was used to determine the time-kill curves for each antibacterial agent alone (at concentration of 1/2 × MIC, MIC and 2 × MIC) and in combination, in the 96 micro-dilution plates. Bacterial counts were taken at 0, 3, 6, 9, and 24 h of incubation with samples plated on BA plates using a 10-fold serial dilution.

After the incubation at 37 °C, colony-forming units (CFUs) were calculated. Synergy between a cell wall synthesis inhibitor and a protein synthesis inhibitor was defined as a ≥ 2 log_10_ CFU/mL reduction in bacterial count by the antibiotic combination compared with the most active single agent after 24 h of treatment. Two treatment conditions were evaluated: (1) monotherapy using MIC values from single-drug testing, and (2) combination therapy using MIC values based on reduced MICs from checkerboard assays. To ensure biological relevance, the combination also had to achieve a ≥ 1 log_10_ CFU/mL reduction compared to the initial bacterial count at 0 h (baseline). A bactericidal effect was defined as a ≥ 3 log_10_ CFU/mL reduction compared with the baseline within 24 h [43].

## 5. Conclusions

*S. suis* remains a major bacterial pathogen in swine, causing severe systemic infections that lead to substantial economic losses and sustained antibiotic use, thereby contributing to growing concerns over antibiotic resistance. This study highlighted the potential of antibiotic combination as an effective approach to combat MDR *S. suis* infection in pig isolates. By combining cell wall synthesis inhibitors (PEN and AMP) with protein synthesis inhibitors (GEN and NEO), significant reductions in the MIC values were achieved, demonstrating synergistic effects. The PEN-GEN combination exhibited the most promising results, with time-kill assays confirming its rapid bactericidal action. These findings supported the ongoing exploration of combination therapies as a strategy to tackle the growing issue of antibiotic resistance. However, careful use of antibiotics is critical to prevent further selection of resistant strains, and additional clinical trials are needed to validate the therapeutic potential of these combinations.

## Figures and Tables

**Table 1:** Distribution of minimum inhibitory concentrations (MICs) for multidrug-resistant *S. suis* isolates (*n* = 36). Green and red vertical lines indicate the susceptible and resistant clinical breakpoints, respectively, based on guidelines from CLSI [15], FDA [16], and previously reported data [17,18,19]. MIC values were interpreted as susceptible (S), intermediate (I), and resistant (R). MIC_50_ and MIC_90_ refer to the MIC values that inhibit 50% and 90% of the isolates, respectively. ND indicates no data.

Antibiotic Drugs	MIC Breakpoints (µg/mL)			MIC Values (µg/mL)																	MIC_50_	MIC_90_	S (%)	I (%)	R (%)	MIC Ranges
	S	I	R	0.02	0.03	0.06	0.125	0.25	0.5	1	2	4	8	16	32	64	128	256	512	1024						
Amoxicillin/Clavulanic acid	≤2/1	4/2	≥8/4		5	9	6	2	1	4	2	2	1		3	1					0.125	32	80.6	5.6	13.9	0.03–64
Ampicillin	≤0.5	1	≥2		1	8	9	1		5	4	2	2			3	1				0.125	64	52.8	13.9	33.3	0.03–128
Penicillin	≤0.25	0.5	≥1		4	2	3	7	7	4	4	1	1	3							0.5	8	44.4	19.4	36.1	≤0.03–16
Vancomycin	≤1	-	≥2					36													0.25	0.25	100.0	0.0	0.0	0.25
Gentamicin	≤4	8	≥16						2	1	3	2	6	1	3	1	5		6	6	32	1024	22.2	16.7	61.1	0.5–1024
Neomycin	≤16	-	≥32							1	3	5	5	5	5	3	4	2	3		16	256	52.8	ND	47.2	1–512
Tilmicosin	≤16	-	≥32										3				3	5	25		512	512	8.3	ND	91.7	8–512
Tylosin tartrate	≤4	-	≥8				1									1		13	21		512	512	2.8	ND	97.2	0.125–512

**Table 2:** Fractional inhibitory concentration indexes (∑FIC) of cell wall synthesis inhibitor penicillin (PEN) in combination with protein synthesis inhibitors gentamicin (GEN), neomycin (NEO), tylosin (TYL), and tilmicosin (TMS) against multidrug-resistant *S. suis* isolates, determined using the microbroth dilution checkerboard method.

Number of Tested Strains	Combined Antibiotics	MIC in Mono (µg/mL)	MIC in Combo (µg/mL)	MIC Fold Change (Mono/Combo) †	ΣFIC	ΣFIC Interpretations				Susceptible Pattern		Number of Tested Strains	ΣFIC Interpretations			
						Synergism	Partial Synergism	Indifference	Antagonism	PEN	Combined Antibiotics		Synergism	Partial Synergism	Indifference	Antagonism
19	GEN	PEN = 0.06–4 GEN = 1–2048	PEN = 0.002–0.25 GEN = 0.25–512	PEN = 4–33 GEN = 4–8	0.25–1.06	10 (52.6%)	7 (36.8%)	2 (10.5%)	0 (0.0%)	NS	NS	6	4 (66.7%)	1 (16.7%)	1 (16.7%)	0 (0.0%)
										NS	S	5	3 (60.0%)	2 (40.0%)	0 (0.0%)	0 (0.0%)
										S	NS	8	3 (37.5%)	4 (50.0%)	1 (12.5%)	0 (0.0%)
22	NEO	PEN = 0.06–32 NEO = 1–1024	PEN = 0.01–8 NEO = 0.25–512	PEN = 4–13 NEO = 4–8	0.19–1.06	7 (31.8%)	9 (40.9%)	6 (27.3%)	0 (0.0%)	NS	NS	4	1 (25.0%)	3 (75.0%)	0 (0.0%)	0 (0.0%)
										NS	S	11	3 (27.3%)	4 (36.4%)	4 (36.4%)	0 (0.0%)
										S	NS	7	3 (42.9%)	2 (28.6%)	2 (28.6%)	0 (0.0%)
23	TYL	PEN = 0.06–16 TYL = 0.25–1024	PEN = 0.03–8 TYL = 0.125–1024	PEN = 4 TYL = 4	0.50–2.00	1 (4.4%)	4 (17.4%)	18 (78.3%)	0 (0.0%)	NS	NS	14	1 (7.1%)	3 (21.4%)	10 (71.4%)	0 (0.0%)
										NS	S	1	0 (0.0%)	0 (0.0%)	1 (100.0%)	0 (0.0%)
										S	NS	8	0 (0.0%)	1 (12.5%)	7 (87.5%)	0 (0.0%)
14	TMS	PEN = 0.125–16 TMS = 8–1024	PEN = 0.01–16 TMS = 0.5–1024	-	0.56–1.08	0 (0.0%)	3 (21.4%)	11 (78.6%)	0 (0.0%)	NS	NS	9	0 (0.0%)	1 (11.1%)	8 (88.9%)	0 (0.0%)
										NS	S	1	0 (0.0%)	0 (0.0%)	1 (100.0%)	0 (0.0%)
										S	NS	4	0 (0.0%)	2 (50.0%)	2 (50.0%)	0 (0.0%)

†: shown only for synergistic cases, NS: non-susceptible, S: susceptible, -: not interpretive.

**Table 3:** Fractional inhibitory concentration indexes (∑FIC) of cell wall synthesis inhibitor ampicillin (AMP) in combination with protein synthesis inhibitors gentamicin (GEN), neomycin (NEO), tylosin (TYL), and tilmicosin (TMS) against multidrug-resistant *S. suis* isolates, determined using the microbroth dilution checkerboard method.

Number of Tested Strains	Combined Antibiotics	MIC in Mono (µg/mL)	MIC in Combo (µg/mL)	MIC Fold Change (Mono/Combo) †	ΣFIC	ΣFIC Interpretations				Susceptible Pattern		Number of Tested Strains	ΣFIC Interpretations			
						Synergism	Partial Synergism	Indifference	Antagonism	AMP	Combined Antibiotics		Synergism	Partial Synergism	Indifference	Antagonism
22	GEN	AMP = 0.03–32 GEN = 1–2048	AMP = 0.004–32 GEN = 0.25–1024	AMP = 3–13 GEN = 4–16	0.19–1.63	5 (22.7%)	9 (40.9%)	8 (36.4%)	0 (0.0%)	NS	NS	7	3 (42.9%)	2 (28.6%)	2 (28.6%)	0 (0.0%)
										NS	S	2	0 (0.0%)	0 (0.0%)	2 (100.0%)	0 (0.0%)
										S	NS	13	2 (15.4%)	7 (53.8%)	4 (30.8%)	0 (0.0%)
20	NEO	AMP = 0.03–64 NEO = 0.5–1024	AMP = 0.004–32 NEO = 0.06–256	AMP = 2–16 NEO = 4–64	0.31–1.03	8 (40.0%)	10 (50.0%)	2 (10.0%)	0 (0.0%)	NS	NS	2	1 (50.0%)	0 (0.0%)	1 (50.0%)	0 (0.0%)
										NS	S	13	4 (30.8%)	8 (61.5%)	1 (7.7%)	0 (0.0%)
										S	NS	5	3 (60.0%)	2 (40.0%)	0 (0.0%)	0 (0.0%)
10	TYL	AMP = 0.5–4 TYL = 256–1024	AMP = 0.06–32 TYL = 16–1024	AMP = 4 TYL = 4	0.5–1.50	1 (10.0%)	2 (20.0%)	7 (70.0%)	0 (0.0%)	NS	NS	8	1 (12.5%)	2 (25.0%)	5 (62.5%)	0 (0.0%)
										S	NS	2	0 (0.0%)	0 (0.0%)	2 (100.0%)	0 (0.0%)
15	TMS	AMP = 0.06–64 TMS = 4–1024	AMP = 0.008–32 TMS = 0.25–512	AMP = 4–8 TMS = 8–32	0.25–1.06	3 (20.0%)	7 (46.7%)	5 (33.3%)	0 (0.0%)	NS	NS	9	2 (22.2%)	4 (44.4%)	3 (33.3%)	0 (0.0%)
										NS	S	3	1 (33.3%)	1 (33.3%)	1 (33.3%)	0 (0.0%)
										S	NS	3	0 (0.0%)	2 (66.7%)	1 (33.3%)	0 (0.0%)

†: shown only for synergistic cases, NS: non-susceptible, S: susceptible.

**Table 4:** Fractional inhibitory concentration indexes (∑FIC) of cell wall synthesis inhibitor amoxicillin/clavulanic acid (AMC) in combination with protein synthesis inhibitors gentamicin (GEN), neomycin (NEO), tylosin (TYL), and tilmicosin (TMS) against multidrug-resistant *S. suis* isolates, determined using the microbroth dilution checkerboard method.

Number of Tested Strains	Combined Antibiotics	MIC in Mono (µg/mL)	MIC in Combo (µg/mL)	MIC Fold Change (Mono/Combo) †	ΣFIC	ΣFIC Interpretations				Susceptible Pattern		Number of Tested Strains	ΣFIC Interpretations			
						Synergism	Partial Synergism	Indifference	Antagonism	AMC	Combined Antibiotics		Synergistic	Partial Synergism	Indifference	Antagonism
21	GEN	AMC = 0.03–32 GEN = 2–2048	AMC = 0.002–8 GEN = 0.5–1024	AMC = 4–33 GEN = 4	0.28–1.31	5 (23.8%)	10 (47.6%)	6 (28.6%)	0 (0.0%)	NS	NS	8	3 (37.5%)	3 (37.5%)	2 (25.0%)	0 (0.0%)
										NS	S	3	1 (33.3%)	1 (33.3%)	1 (33.3%)	0 (0.0%)
										S	NS	10	1 (10.0%)	6 (60.0%)	3 (30.0%)	0 (0.0%)
19	NEO	AMC = 0.03–32 NEO = 2–2048	AMC = 0.002–16 NEO = 0.5–512	AMC = 4–15 NEO = 4–16	0.30–1.00	4 (21.1%)	12 (63.2%)	3 (15.8%)	0 (0.0%)	NS	S	8	1 (12.5%)	7 (87.5%)	0 (0.0%)	0 (0.0%)
										S	NS	11	3 (27.3%)	5 (45.5%)	3 (27.3%)	0 (0.0%)
10	TYL	AMC = 0.5–32 TYL = 0.25–1024	AMC = 0.03–16 TYL = 0.125–1024	-	0.53–1.50	0 (0.0%)	4 (40.0%)	6 (60.0%)	0 (0.0%)	NS	NS	7	0 (0.0%)	3 (42.9%)	4 (57.1%)	0 (0.0%)
										NS	S	1	0 (0.0%)	0 (0.0%)	1 (100.0%)	0 (0.0%)
										S	NS	2	0 (0.0%)	1 (50.0%)	1 (50.0%)	0 (0.0%)
20	TMS	AMC = 0.06–64 TMS = 8–1024	AMC = 0.004–32 TMS = 4–1024	-	0.56–1.50	0 (0.0%)	2 (10.0%)	18 (90.0%)	0 (0.0%)	NS	NS	11	0 (0.0%)	2 (18.2%)	9 (81.8%)	0 (0.0%)
										NS	S	1	0 (0.0%)	0 (0.0%)	1 (100.0%)	0 (0.0%)
										S	NS	8	0 (0.0%)	0 (0.0%)	8 (100.0%)	0 (0.0%)

†: shown only for synergistic cases, NS: non-susceptible, S: susceptible, -: not interpretive.

**Table 5:** Fractional inhibitory concentration indexes (∑FIC) of cell wall synthesis inhibitor vancomycin (VAN) in combination with protein synthesis inhibitors gentamicin (GEN), neomycin (NEO), tylosin (TYL), and tilmicosin (TMS) against multidrug-resistant *S. suis* isolates, determined using the microbroth dilution checkerboard method.

Number of Tested Strains	Combined Antibiotics	MIC in Mono (µg/mL)	MIC in Combo (µg/mL)	MIC Fold Change (Mono/Combo) †	ΣFIC	ΣFIC Interpretations				Susceptible Pattern		Number of Tested Strains	ΣFIC Interpretations			
						Synergism	Partial Synergism	Indifference	Antagonism	VAN	Combined Antibiotics		Synergism	Partial Synergism	Indifference	Antagonism
4	GEN	VAN = 0.125–0.25 GEN = 512–1024	VAN = 0.02–0.2 GEN = 32–1024	-	0.66–1.16	0 (0.0%)	2 (50.0%)	2 (50.0%)	0 (0.0%)	S	NS	4	0 (0.0%)	2 (50.0%)	2 (50.0%)	0 (0.0%)
3	NEO	VAN = 0.125–0.25 NEO = 128–1024	VAN = 0.02–0.125 NEO = 32–1024	-	0.66–1.16	0 (0.0%)	1 (33.3%)	2 (66.7%)	0 (0.0%)	S	NS	3	0 (0.0%)	1 (33.3%)	2 (66.7%)	0 (0.0%)
4	TYL	VAN = 0.25 TYL = 512	VAN = 0.25 TYL = 16	-	1.03	0 (0.0%)	0 (0.0%)	4 (100.0%)	0 (0.0%)	S	NS	4	0 (0.0%)	0 (0.0%)	4 (100.0%)	0 (0.0%)
4	TMS	VAN = 0.125–0.25 TMS = 512–1024	VAN = 0.125–0.25 TMS = 16–32	-	1.03	0 (0.0%)	0 (0.0%)	4 (100.0%)	0 (0.0%)	S	NS	4	0 (0.0%)	0 (0.0%)	4 (100.0%)	0 (0.0%)

†: shown only for synergistic cases, NS: non-susceptible, S: susceptible, -: not interpretive.

## Data Availability

The authors confirm that the data supporting the findings of this study are available within the articles and Supplement Materials. Further inquiries can be directed to the corresponding authors.

## Supplementary Materials

The following supporting information can be downloaded at: https://www.mdpi.com/article/10.3390/antibiotics15020136/s1, Table S1: Combination MICs and FICI values of penicillin (PEN) plus protein synthesis inhibitors gentamicin (GEN), neomycin (NEO), tylosin (TYL), and tilmicosin (TMS) against multidrug-resistant (MDR) *S. suis* isolates, determined from microbroth dilution checkerboard tests; Table S2: Combination MICs and FICI values of ampicillin (AMP) plus protein synthesis inhibitors gentamicin (GEN), neomycin (NEO), tylosin (TYL), and tilmicosin (TMS) against multidrug-resistant (MDR) *S. suis* isolates, determined from microbroth dilution checkerboard tests; Table S3: Combination MICs and FICI values of amoxicillin/clavulanic acid (AMC) plus protein synthesis inhibitors gentamicin (GEN), neomycin (NEO), tylosin (TYL), and tilmicosin (TMS) against multidrug-resistant (MDR) *S. suis* isolates, determined from microbroth dilution checkerboard tests; Table S4: Combination MICs and FICI values of vancomicin (VAN) plus protein synthesis inhibitors gentamicin (GEN), neomycin (NEO), tylosin (TYL), and tilmicosin (TMS) against multidrug-resistant (MDR) *S. suis* isolates, determined from microbroth dilution checkerboard tests; Table S5: MIC breakpoints and interpretative categories of antimicrobial susceptibility test; Figure S1: Overview of the MDR *S. suis* isolates (*n* = 36) tested with 16 paired-antibiotic combinations using the microbroth dilution checkerboard method. Colored cells indicate the specific MDR *S. suis*-drug pair combinations evaluated, with I, P, and S denoting indifference (orange), partial synergism (light green), and synergism (dark green), respectively.
