# Supplementary figures and images for: In Vitro Synergistic Effects of Antibiotic Combinations Against Multidrug-Resistant Streptococcus suis from Diseased Pigs
Source: Antibiotics (Basel). 2026 Jan 29;15(2):136. doi: 10.3390/antibiotics15020136 (PMC12937455; doi:10.3390/antibiotics15020136)

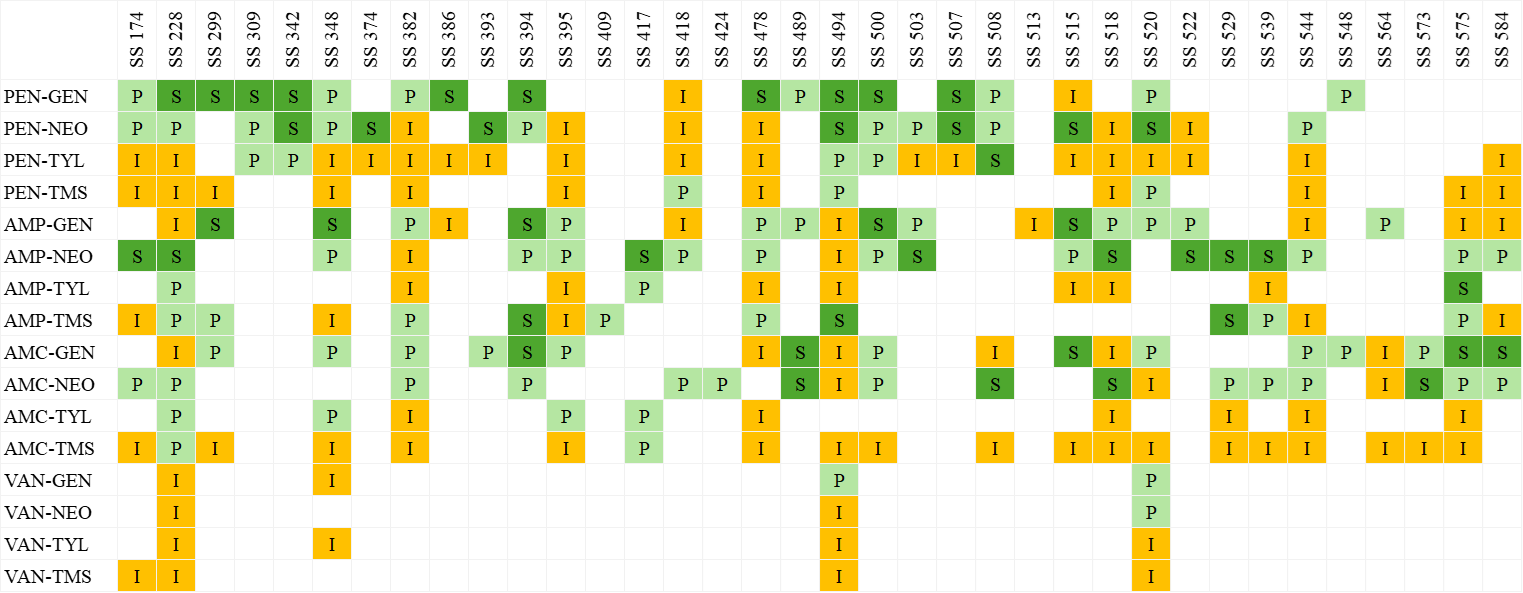

Supplement: Supplementary file 1 [file antibiotics-15-00136-s001.zip › Supplementary Fig. S1_Chumpol W_24OCT2025_SY.tif]
